# Tracking government spending on immunization*:* The joint reporting forms, national health accounts, comprehensive multi-year plans and co-financing data

**DOI:** 10.1016/j.vaccine.2021.04.047

**Published:** 2021-06-08

**Authors:** Gloria Ikilezi, Steven D Bachmeier, Ian E Cogswell, Emilie R Maddison, Hayley N Stutzman, Golsum Tsakalos, Logan Brenzel, Joseph L Dieleman, Angela E Micah

**Affiliations:** a Gates Ventures, United States; b Institute for Health Metrics and Evaluation, United States; c Bill & Melinda Gates Foundation, United States

**Keywords:** Immunization spending, Government spending, Joint Reporting Form, National Health Accounts

## Abstract

**Background:**

Coverage rates for immunization have dropped in lower income countries during the COVID-19 pandemic, raising concerns regarding potential outbreaks and premature death. In order to re-invigorate immunization service delivery, sufficient financing must be made available from all sources, and particularly from government resources. This study utilizes the most recent data available to provide an updated comparison of available data sources on government spending on immunization.

**Methods:**

We examined data from WHO/UNICEF’s Joint Reporting Form (JRF), country Comprehensive Multi-Year Plan (cMYP), country co-financing data for Gavi, and WHO National Health Accounts (NHA) on government spending on immunization for consistency by comparing routine and vaccine spending where both values were reported. We also examined spending trends across time, quantified underreporting and utilized concordance analyses to assess the magnitude of difference between the data sources.

**Results:**

Routine immunization spending reported through the cMYP was nearly double that reported through the JRF (*rho* = 0.64, 95% 0.53 to 0.77) and almost four times higher than that reported through the NHA on average (*rho* = 3.71, 95% 1.00 to 13.87). Routine immunization spending from the JRF was comparable to spending reported in the NHA (*rho* = 1.30, 95% 0.97 to 1.75) and vaccine spending from the JRF was comparable to that from the cMYP data (*rho* = 0.97, 95% 0.84 to 1.12). Vaccine spending from both the JRF and cMYP was higher than Gavi co-financing by a at least two (*rho* = 2.66, 95% 2.45 to 2.89) and (*rho* = 2.66, 95% 2.15 to 3.30), respectively.

**Implications:**

Overall, our comparative analysis provides a degree of confidence in the validity of existing reporting mechanisms for immunization spending while highlighting areas for potential improvements. Users of these data sources should factor these into consideration when utilizing the data. Additionally, partners should work with governments to encourage more reliable, comprehensive, and accurate reporting of vaccine and immunization spending.

## 1 Introduction

Tracking resources spent on immunization is important for understanding spending patterns and for monitoring progress towards global goals, such as the goals and targets set by the Global Vaccine Action Plan and the Immunization Agenda 2030 [1], [2]. The GVAP articulated the actions needed to realize the vision cast by the Decade of Vaccines that all individuals have lives free from vaccine preventable diseases [2], [3]. The plan used the indicator of government spending on immunization as a measure of country progress on domestic ownership and commitment to the immunization agenda. This interest in monitoring domestic ownership of the immunization agenda has also been advanced through the co-financing requirement policies by Gavi, the Vaccine Alliance. This co-financing policy requires country partners to also contribute some financial resources which are determined based on income towards the procurement of their vaccines [4]. Globally, there are four main sources of data for understanding the spending of governments towards immunization. These are WHO/UNICEF’s Joint Reporting Form (JRF), the country Comprehensive Multi Year Plan (cMYP), country co-financing data through UNICEF procurement and WHO National Health Accounts (inclusive of System Health Accounts, National Health Accounts and the Global Health Expenditure Database) [4], [5], [6], [7]. The process for collating the data is distinct for each data source. For instance, for the Joint Reporting Form, it is a collaboration between UNICEF and WHO and also involves country counterparts while the co-financing data is obtained from data on country contributions as recorded in procurement databases.

Despite the availability of these sources of data on government spending on immunization, there are concerns regarding the reliability and completeness of these estimates of government spending on immunization [8], [9]. A report from the WHO in 2017 that analyzed the financing indicators reported in the JRF highlighted the changes over time in reported missingness and inconsistencies in the reported data [8]. Another study by Nader et al reported that the government spending on routine immunization in the JRF was 83 percent what was reported in the corresponding CMYP base year [10]. These previous studies have highlighted the challenges with the available data on government spending on immunization.

This study aims to utilize the most recent data possible to provide an updated comparison of available data sources on government spending on immunization, with a focus on identifying strengths and limitations of the available data. The remainder of the paper is laid out as follows. Section 2 follows with a detailed description of the data sources used in this analysis and the comparative analyses conducted. Section 3 presents the results of our analyses and Section 4 provides the discussion of the key findings, limitations and provide conclusions.

## 2 Methodology

### 2.1 Data sources and definitions

The analyses relied on data from four key sources of government spending data. The data sources were the WHO JRF, country (cMYPs) and Financial Sustainability Plans (FSPs), WHO NHAs and co-financing data [4], [5], [11], [12], [13]. We focused the analyses on low and middle- income countries because it is important to understand how spending in these countries has evolved given Gavi policies of co-financing since 2006. The analyses relied on data from 2000 through 2017. The analyses relied on data from 2000 through 2017. Of the 135 countries included in the study, 70 (51.9%) were Gavi recipient countries, while 46 (34.1%) countries were in Sub-Saharan Africa, 25 (18.5%) countries in the Latin America & Caribbean region, 24 (17.8%) in Southeast Asia, East Asia, and Oceania, 20 (14.8%) in Central Europe, Eastern Europe & Central Asia, 15 (11.1%) in North Africa & Middle East, and 5 (3.7%) in South Asia.

The JRF form which is coordinated by WHO and UNICEF, is distributed to the national immunization program point person in the Ministry of Health for all Member States , at the start of each year. Once completed, the forms are returned and data are extracted by WHO Headquarters, reviewed for completeness and consistency and queries are sent to countries to clarify missingness and inconsistencies [5]. The immunization system performance data are collected for a calendar year, with the flexibility to update prior years’ data at any time through written communication to WHO and/or UNICEF.

From the JRF, we extracted two indicators -- indicator 6540, which reports on government spending on routine immunization including vaccines, and indicator 6510, which reports government spending data on vaccines [11]. Data was available spanning 2006 to 2017 for 132 countries. The former consists of recurrent immunization specific spending for routine immunization excluding shared health system costs, while the latter comprises spending for vaccines including injection supplies excluding those used for supplementary immunization activities. Vaccine expenditures captured through the JRF are a subset of the routine immunization expenditure envelope reported through the same.

The cMYP, on the other hand, is a financial planning framework that uses an Excel-based tool for estimating the costs and financing of immunization for a baseline year, and formulating estimates of resource requirements of the national strategy for a future 5-year period, and quantifying any financing gap [14].

We extracted baseline year data on routine immunization and vaccine specific spending from the cMYP and FSP. This historic base year provides a reference year from which comparisons can be made between current programmatic costs and future resource requirements. Recurrent program spending was collected from this tool excluding reported shared health systems costs. The extracted reported values comprised of government spending on vaccines, Gavi co-financing disbursements as well as operational costs. Vaccine spending from the cMYPs is typically disaggregated into vaccine and injection safety line items, with spending on vaccines further differentiated by those used during routine activities versus those that are administered during supplementary immunization activities. We extracted data from the cMYPs spanning 2000 through 2017 for 72 countries. To make the cMYP data comparable to estimates reported by other data sources, we combined injection supplies with spending on vaccines. In addition, we excluded spending on supplementary vaccines from the cMYPs to align with both JRF vaccine and Gavi co-financing spending definitions.

Gavi co-financing is tracked and monitored by UNICEF and Gavi partners and is linked to country co-procurement of supported vaccines. Details on co-financing requirements by vaccine and procurement to date by the country is monitored and updated at least twice per year to account for new information on co-financing and eligibility status [4]. From the Gavi co-financing reports, we extracted the amount of spending recipient countries commit to spending on new vaccines supported by Gavi, for which available data spans 2008 through 2017. These reported values exclude vaccines administered during supplementary activities which are typically fully financed by Gavi. These reports were available for 70 countries.

For the NHA, country official focal persons are appointed to lead the data production process in a phased manner including data collection, technical review with the WHO, country and international experts, and data validation. Quality checks and data validation are an inherent part of the production of the health accounts estimates including missing data analysis, negative values indication, cross classification comparison, consistency checks, revisions of time and compilation issues. Government spending data are verified and validated by comparison with macroeconomic variables, such as the share of general government expenditure on health [13].

Child health sub-accounts existed between 2003 and 2009 for 2 countries. We extracted all spending values on immunization under the preventive health function from the child health sub-accounts. These captured spending on immunization programs for child health. All together, we extracted 7 country years of data.

We also extracted data from NHA between 2000 and 2017 for 23 countries. Where spending values by the government as the source were unavailable, we extracted spending values from health function 6.2 on immunization programs which represents the spending by government as the financing agency. In the NHAs, spending on various health functions is not disaggregated by financing source such as government, out of pocket and development assistance. For that reason, we utilized the relationship between the reported data on immunization spending and total health spending by government as financing agent and applied that proportion to the reported immunization envelope as illustrated in the equation below.

$$\frac{{\mathrm{government}\;financing\;scheme}_{\mathrm{governmentrevenue}}}{{\mathrm{government}\;financing\;scheme}_{\mathrm{governmentrevenue}+othersources}}*{\mathrm{HC}.6.2}_{\mathrm{governmentfinancingscheme}}$$

In addition, we extracted domestic general government spending on immunization from 2015 to 2017 from the GHED to supplement the NHA data. We assessed the reliability of the estimates of government spending on immunization we calculated using data from the NHA.

All spending estimates were converted to 2019 USD. To convert our estimates, we used country specific deflator series to convert reported values in nominal local currency units to 2019 real local currency units. These real values were then converted to 2019 USD using country specific exchange rates.

### 2.2 Statistical analysis

We explored these data for consistency by comparing routine and vaccine spending where both values were reported for a given country year.

(i) First, we identified data points where vaccine spending exceeded that on routine immunization by assessing the ratio of vaccine to routine spending, which should generally be less than one. Values for which vaccine spending was greater than routine immunization spending were excluded from further analyses.
(ii) Second, we examined spending trends for values across time (when both sources reported for the same country and year). These were reported as spending per surviving infant [15].
(iii) Third, we quantified underreporting, which we defined as zero (or less than $0.001 per surviving infant) estimates or missing JRF data. We did not quantify missingness from the cMYPs or the NHAs since reporting in these data sources vary from country to country. For Gavi co-financing data we did not quantify missingness either, since countries have an opportunity to defer payments to subsequent years where they are obliged to fulfill both current and deferred financing requirements.
(iv) Fourth, for all data sources for which the reported values were comparable, we generated scatter plots with their corresponding correlation coefficients. To quantify the magnitude of existing difference between the different data sources reporting similar spending measures, we ran concordance analyses using the Bland Altman regression method. We used this method to leverage the ability to not only assess the relationship between the data sources, but also to quantify the agreement between the two measures by studying the mean difference and constructing limits of agreement [16]. Analyses were performed using Stata (version 15.1) and R (version 3.2.2).

## 3 Results

The study included 132 countries with 3,712 data points in total, majority of which were derived from tabulated JRF data, and the least from NHA reports.. Table 1 provides additional details on the input data for the study. There were 3 countries for which we did not have reported values in any of the data sources used in the study: West Bank and Gaza (Palestine), Serbia, and American Samoa.

**Table 1:** Data used in the study by source and GBD super region.

**Data source**	**GBD super region**	**Number of countries**	**Number of data points**	**Minimum year**	**Maximum year**	**Minimum**	**Maximum**	**p50**	**Mean**	**Standard deviation**
Gavi co-financing of vaccines	All GBD super regions	70	596	2008	2017	0.1	26	1	2	3
JRF: Government spending on routine immunization	All GBD super regions	129	1215	2006	2017	0.27	1657	19	48	85
JRF: Government spending on vaccines	All GBD super regions	131	1374	2006	2017	0.07	690	13	37	60
SHA/GHED: Government spending on immunization programs	All GBD super regions	55	164	2000	2017	0.002	735	11	58	111
cMYP/FSP: Government spending on routine immunization	All GBD super regions	72	239	2000	2017	0.05	179	8	14	20
cMYP/FSP: Government spending on vaccines	All GBD super regions	62	124	2000	2017	0.11	36	3	5	6
Gavi co-financing of vaccines	Central Europe, Eastern Europe, and Central Asia	8	68	2008	2017	0.36	24	2	4	5
JRF: Government spending on routine immunization	Central Europe, Eastern Europe, and Central Asia	18	135	2006	2017	0.75	451	35	67	88
JRF: Government spending on vaccines	Central Europe, Eastern Europe, and Central Asia	19	198	2006	2017	0.19	690	33	65	92
SHA/GHED: Government spending on immunization programs	Central Europe, Eastern Europe, and Central Asia	8	17	2014	2017	0.72	260	69	86	93
cMYP/FSP: Government spending on routine immunization	Central Europe, Eastern Europe, and Central Asia	11	32	2000	2014	0.91	104	19	27	23
cMYP/FSP: Government spending on vaccines	Central Europe, Eastern Europe, and Central Asia	9	19	2000	2014	0.99	36	7	12	11
Gavi co-financing of vaccines	Latin America and Caribbean	5	41	2008	2017	0.19	20	3	5	5
JRF: Government spending on routine immunization	Latin America and Caribbean	25	273	2006	2017	0.36	1657	81	111	132
JRF: Government spending on vaccines	Latin America and Caribbean	24	272	2006	2017	6	381	57	78	60
SHA/GHED: Government spending on immunization programs	Latin America and Caribbean	4	13	2011	2017	25	275	39	74	77
cMYP/FSP: Government spending on routine immunization	Latin America and Caribbean	2	3	2000	2006	0.65	83	1	28	47
Gavi co-financing of vaccines	North Africa and Middle East	3	27	2008	2017	0.19	4	1	1	1
JRF: Government spending on routine immunization	North Africa and Middle East	14	121	2006	2017	0.46	411	23	52	70
JRF: Government spending on vaccines	North Africa and Middle East	14	139	2006	2017	0.22	385	24	48	62
SHA/GHED: Government spending on immunization programs	North Africa and Middle East	2	4	2014	2017	1	70	4	20	34
cMYP/FSP: Government spending on routine immunization	North Africa and Middle East	3	16	2001	2013	0.05	27	8	11	10
cMYP/FSP: Government spending on vaccines	North Africa and Middle East	3	7	2001	2013	0.29	6	3	3	2
Gavi co-financing of vaccines	South Asia	4	35	2008	2017	0.4	7	1	2	1
JRF: Government spending on routine immunization	South Asia	5	58	2006	2017	3	98	8	12	17
JRF: Government spending on vaccines	South Asia	5	58	2006	2017	0.4	11	4	5	3
SHA/GHED: Government spending on immunization programs	South Asia	5	27	2000	2017	0.002	56	1	7	13
cMYP/FSP: Government spending on routine immunization	South Asia	5	16	2001	2012	2	44	16	19	12
cMYP/FSP: Government spending on vaccines	South Asia	5	8	2001	2012	0.56	4	3	3	1
Gavi co-financing of vaccines	Southeast Asia, East Asia, and Oceania	11	85	2008	2017	0.36	17	2	3	3
JRF: Government spending on routine immunization	Southeast Asia, East Asia, and Oceania	23	209	2006	2017	0.43	277	18	25	31
JRF: Government spending on vaccines	Southeast Asia, East Asia, and Oceania	23	235	2006	2017	0.43	292	12	24	36
SHA/GHED: Government spending on immunization programs	Southeast Asia, East Asia, and Oceania	7	21	2007	2017	1	348	239	158	134
cMYP/FSP: Government spending on routine immunization	Southeast Asia, East Asia, and Oceania	9	25	2000	2017	0.07	67	8	13	16
cMYP/FSP: Government spending on vaccines	Southeast Asia, East Asia, and Oceania	9	15	2001	2017	0.18	25	7	8	6
Gavi co-financing of vaccines	Sub-Saharan Africa	39	340	2008	2017	0.1	26	1	2	2
JRF: Government spending on routine immunization	Sub-Saharan Africa	44	419	2006	2017	0.27	224	6	17	32
JRF: Government spending on vaccines	Sub-Saharan Africa	46	472	2006	2017	0.07	219	3	10	27
SHA/GHED: Government spending on immunization programs	Sub-Saharan Africa	29	82	2003	2017	0.007	735	8	43	117
cMYP/FSP: Government spending on routine immunization	Sub-Saharan Africa	42	147	2000	2016	0.06	179	6	11	21
cMYP/FSP: Government spending on vaccines	Sub-Saharan Africa	36	75	2000	2016	0.11	17	2	3	3

Routine immunization spending was predominantly reported through the JRF at 32.7% (1215 data points) compared to 6.4% (239 data points) and 4.4% (164 data points from the cMYPs and NHA respectively). Vaccine spending data overlapped with 1,374 data points (37.0%) and 124 data points (3.3%) from the JRF and cMYP respectively.

From available JRF routine immunization data, we matched 85 data points to those reported through cMYP and 135 through the NHA. We had the highest overlap between JRF vaccine and Gavi co-financing data with 563 data points and the least between cMYP routine immunization spending and NHA data with only 13 data points. From the cMYP vaccine data, 78 data points were matched to JRF vaccine between 2006 and 2017 and 68 to Gavi co-financing data between 2008 and 2017.

From the JRF, the median government spending per surviving infant on routine immunization was

$19.14 and $12.74 on vaccines ranging from $0.27 to $1657 and $0.07 to $690 respectively.

Government spending on routine immunization per surviving infant varied substantially by region from

$81.00 (range; $0.36-$1657) in Latin America and Caribbean to USD $5.92 ($0.27-$224) in sub-Saharan Africa. The highest vaccine spending by government was reported in Latin America and Caribbean regions at $56.99 ($6.01-$381) and lowest in sub-Saharan Africa $3.23 ($0.07-$219).

By contrast, reported government spending on total immunization from the NHA data (median $10.61 ($0.002-$735)) was about one half of the reported JRF spending and even lower in the government spending on routine immunization data from the cMYP with a median of $8.09 ($0.05-$179)). We observed a similar pattern with government vaccine spending values from the JRF remaining substantially higher compared to that reported through the cMYPs overall. Across regions, this pattern held except for sub-Saharan where government vaccine spending from both sources were comparable as indicated in Table 1.

Gavi co-financing reported values were lower than reported government vaccine spending values from

the JRF and cMYP data, with less regional variability ranging from $2.97 in Latin America and Caribbean,

$1.69 in Central Europe and South East Asia and $1.19 in all other regions.

**Fig. 1:**
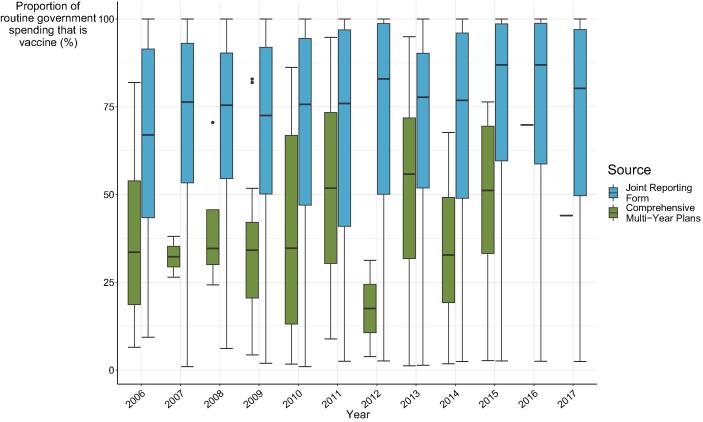
The proportion of routine government spending that is spending on vaccines for the Joint Reporting Form (JRF) and Comprehensive Multi-Year Plans (cMYP). Note: Values of zero (and values less than $0.001 per surviving infant) were removed as missingness. The boxplots presenting Comprehensive Multi-Year Plans for 2008, 2016, and 2017 are representative of only 5 data points (1 outlier), 1 data point, and 1 data point, respectively, resulting in distorted boxplots.

Based on the JRF from which we had majority of the data points, the lowest levels of government spending for both routine immunization and vaccines were reported in South East Asia and sub-Saharan Africa. Fig. 1 highlights the spread of data points on the share of routine government spending that is spent on vaccines as reported in the JRF and the cMYP from 2006 through 2017. The median proportion of routine government spending that is vaccine reported in the JRF is higher than that reported in the cMYP over the entire period with data. The median from the JRF ranges from 66.9% to 86.9% whereas that reported in the cMYP ranges from 17.5% to 55·8% for the years with at least two data points. Across most years, the JRF also reports as minimum proportion a value of close to zero and a maximum proportion value of 100%. While there are multiple instances in which a minimum value close to zero is reported in the cMYP, the maximum proportion of 100% is never reported in the cMYP over the period 2006 through 2017. Lastly, most data points from the JRF are concentrated between the median and 100% whereas the data points from the cMYP have no such distinct tendency.

**Fig. 2:**
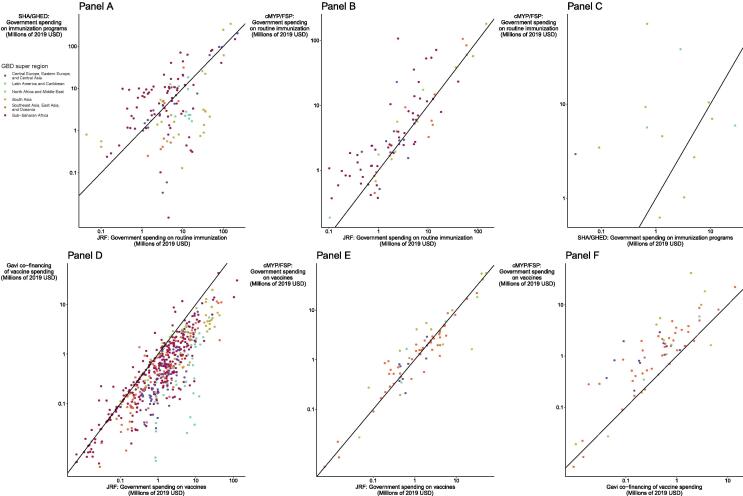
Comparison of raw data between each source for spending on vaccines or routine immunization. Note: Data are presented in inflation-adjusted 2019 US$. Colors represent GBD super regions. A line of equality is plotted on each panel.

Fig. 2 presents a series of figures that compare reported values of government spending on routine immunization and vaccines across the different data sources included in these analyses. Overall, large reported values in one data source are aligned with large reported values in the comparison data source. For example, in panel A, when JRF reported values of government spending on routine immunization are compared to reported values of government immunization spending values from the NHA, there seems to be a clustering of instances when reported values in the JRF is larger while there is a more distributed spread of instances when the reported values from the NHA are larger. When comparing the government spending on routine immunization between JRF and cMYP (panel B), more of the data points reported in the cMYP are larger than those reported in the JRF.

We also compared the JRF vaccine envelope to the Gavi co-financing spending for 68 Gavi recipient countries for which we had overlapping data between 2008 and 2017 Fig. 3. Overall, we observed a positive correlation of 0.74 with substantial variation across countries ranging from 0.99 in Uzbekistan to −0.78 in Malawi. Fifteen countries had a negative correlation coefficient the lowest of which included Guinea, Kenya, Djibouti, and Honduras. Other countries with a strong correlation of greater than 0.9 included Bhutan, Sudan, Timor-Leste, Afghanistan, Mauritania and Mozambique. A further analysis of the proportions of Gavi co-financing on the government spending on vaccine reported by JRF by country and by year is reported in the annex Table 3. By country and year, we observed instances where the reported co-financing value is much larger than that in the JRF.

**Fig. 3:**
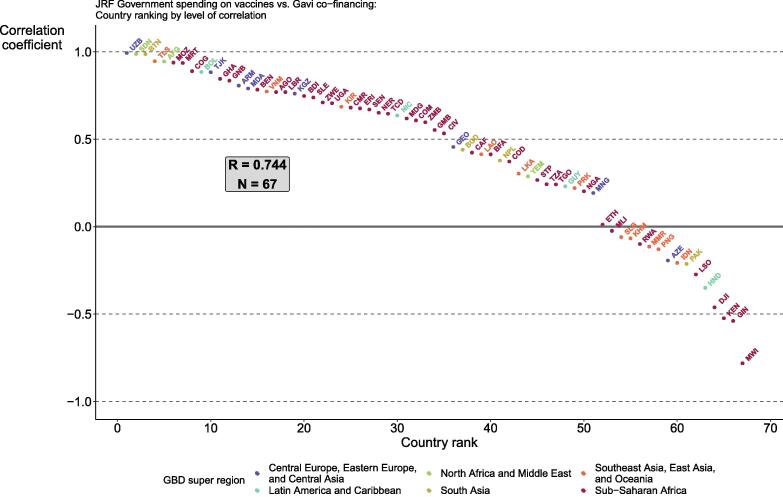
Correlation coefficients of spending on vaccines between the Joint Reporting Form (JRF) and Gavi co-financing values, ranked by country in decreasing order. Note: Colors represent GBD super regions. AFG = Afghanistan. AGO = Angola. ARM = Armenia. AZE = Azerbaijan. BEN = Benin. BDI = Burundi. BFA = Burkina Faso. BGD = Bangladesh. BOL = Bolivia. BTN = Bhutan. CAF = Central African Republic. CIV = Côte d'Ivoire. CMR = Cameroon. COD = DR Congo. COG = Congo (Brazzaville). COM = Comoros. DJI = Djibouti. ERI = Eritrea. ETH = Ethiopia. GEO = Georgia. GHA = Ghana. GIN = Guinea. GMB = The Gambia. GNB = Guinea-Bissau. GUY = Guyana. HND = Honduras. IDN = Indonesia. KEN = Kenya. KGZ = Kyrgyzstan. KHM = Cambodia. KIR = Kiribati. LAO = Laos. LBR = Liberia. LKA = Sri Lanka. LSO = Lesotho. MDA = Moldova. MDG = Madagascar. MLI = Mali. MMR = Myanmar. MNG = Mongolia. MOZ = Mozambique. MRT = Mauritania. MWI = Malawi. NER = Niger. NIC = Nicaragua. NGA = Nigeria. NPL = Nepal. PAK = Pakistan. PNG = Papua New Guinea. PRK = North Korea. RWA = Rwanda. SDN = Sudan. SEN = Senegal. SLB = Solomon Islands. SLE = Sierra Leone. STP = São Tomé and PrÍncipe. TCD = Chad. TGO = Togo. TJK = Tajikistan. TLS = Timor-Leste. TZA = Tanzania. UGA = Uganda. UZB = Uzbekistan. VNM = Vietnam. YEM = Yemen. ZMB = Zambia. ZWE = Zimbabwe.

**Fig. 4:**
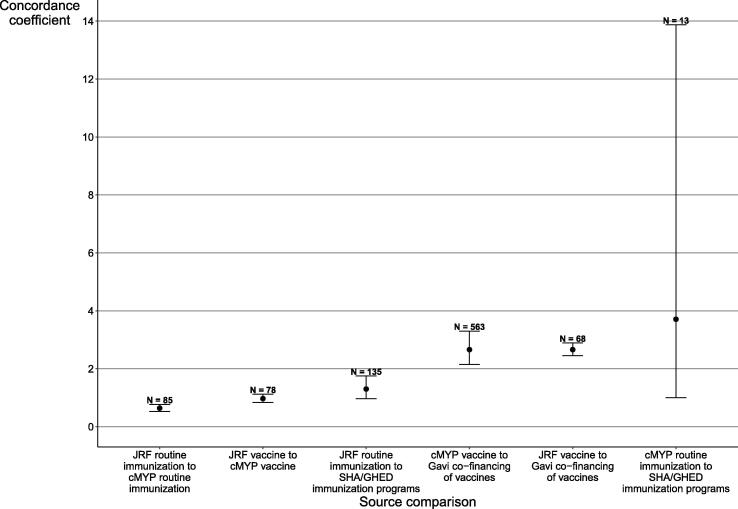
Concordance coefficients between each source of data for spending on vaccines or routine immunization. Note: Error bars represent two standard deviations from the coefficient. Number of data points is labeled above the upper error bar.

Results from the concordance analyses highlight variations in the magnitude of average differences across data sources measuring similar components for a given country year Fig. 4. Routine immunization spending reported through the cMYP was close to double that reported through the JRF (*rho* = 0.64, 95% 0.53 to 0.77) and almost four times higher than that reported through the NHA on average (*rho* = 3.71, 95% 1.00 to 13.87).

On the other hand, routine immunization spending by government from the JRF was comparable to government spending reported in the NHA (*rho* = 1.30, 95% 0.97 to 1.75) and vaccine spending from the JRF was comparable to that from the cMYP data (*rho* = 0.97, 95% 0.84 to 1.12). As expected, vaccine spending from both the JRF and cMYP was higher than Gavi co-financing by a magnitude of at least 2 (*rho* = 2.66, 95% 2.45 to 2.89) and (*rho* = 2.66, 95% 2.15 to 3.30) respectively. Table 1 in the annex provides additional detail on the specific cost components included in the government spending on routine immunization from each data source.

## 4 Discussion

Our analysis is based on 135 low- and middle- income countries between 2000 and 2017. This timeframe overlaps with the latter half of the Global Vaccine Action Plan, which emphasized the need for improved accountability as national investments continue to grow. Currently, the Immunization Agenda 2030 (IA2030) draws on lessons learnt over the last ten years with one of the key objectives being efficient use of resources for self-sustainability as well as the use of high-quality, “fit-for-purpose” data for action at all levels [17]. Accuracy, consistency and completeness in reporting mechanisms are critical in achieving this objective especially as countries continue to adopt new vaccines and transition towards self-financing.

This study leverages various data sources for reporting government spending on routine immunization. These data are designed for different functions; for instance, the JRF mechanism is primarily used by WHO Member States to report country immunization spending and Gavi co-financing to assess recipient country trajectories towards increased ownership of vaccine financing and overall financial sustainability. Despite these functional differences, reporting structures remain mostly comparable by line item providing an opportunity for benchmarking and quality checks to inform areas of improvement and innovation where necessary.

Our analysis shows incompleteness for government routine immunization spending reported though the JRF was higher than that for reported vaccine data. This may be due to the augmented intricacies in collating spending on all routine components compared to vaccines which are mostly standard with streamlined data sources [18], [19]. Under reporting was particularly high in thirteen countries where both spending categories (vaccine and routine) were missing for at least half the timeframe of our study period. These varied by income level and geographical location pointing to differences in health system capacity as well as varying accountability mechanisms across reporting countries. This could potentially lead to selection bias, which may have under or overestimated the different spending values. However, where reporting was complete for both line items, we only found three implausible data points where vaccine spending exceeded routine immunization from the JRF and none from the cMYPs. Overall, this suggests some degree of accuracy where missingness is not an issue, but on the other hand calls for improved data quality checks particularly in the thirteen countries mentioned earlier.

We found high comparability between vaccine spending data reported through the JRF and baseline year cMYPs, and between routine immunization spending reported through the JRF and NHA data. This finding suggests consistency in reporting mechanisms for these, also highlighting the importance of standardizing source data for the different line items. While vaccine data from the two sources – JRF and cMYPs- appeared aligned, routine immunization was substantially lower in the JRF compared to the cMYPs likely due to the inclusion of routine capital costs, some of which are met by governments. While government contributions where present are included in the cMYP envelope, the JRF consistently captures the routine recurrent costs.

Unsurprisingly, vaccine spending from both cMYP and JRF exceeded Gavi co-financing which captures spending on vaccines supported by Gavi alone as opposed to the two sources which are made up of spending on all traditional and new vaccines provided through the national Expanded Program on Immunization (EPI) programs. Given that Gavi co-financing spending is included in the overall vaccine spending reported through the JRF, both envelopes should increase uniformly reflecting additional government investments on vaccines. Our correlation analysis however showed an inverse correlation between the two data sources in fifteen countries with the lowest correlation in Lesotho, Honduras, Guinea, Kenya and Malawi. This calls for more rigorous reporting mechanisms among countries especially as their financial obligations grow towards transition and self-financing.

Furthermore, there were some observed instances when reported values in the JRF is larger than the reported values from the NHA. This is surprising when we assume that the NHA are inclusive of other system costs that may not be captured in the JRF.

Our results show that the median spending for routine immunization varied from $19 in the JRF data to $11 and $8 per the NHA and cMYP data, respectively. These values varied by region with similar patterns inherently due to the disproportionate number of data points across the different regions. For instance, 61% of all data points on baseline cMYP data were from sub-Saharan Africa suggesting the reported spending was skewed to countries of similar income levels. In sub-Saharan Africa alone, the median routine immunization spending reported through the JRF was equal to that reported through the cMYP. While more countries continue to adhere to current reporting practices, this finding underscores the need for improved coverage by country to allow for proportionate representation by geographical region and income level.

This comparison analysis has several limitations. First, the data sources used were generated for different purposes and as such measured slightly different things. Our analyses were restricted to comparisons of the same metrics and excluded estimates such as shared health systems costs and spending on vaccines for supplementary campaigns which were only measured by one data source. Second in some instances the time periods of data available from the different data sources did not overlap in all years and so there were instances where there were gaps in comparison due to the availability of data. Third, in instances in which country reporting were systematically biased due to under-reporting or misreporting the results from our analyses may be skewed. We acknowledge this as a limitation for secondary analyses that is largely dependent on reported values.

The monitoring of targets for the Global Vaccine Action Plan in the last decade has emphasized the importance of having reliable and credible immunization spending data. This study has highlighted the strengths and challenges with the currently available data sources for monitoring spending on immunization. Users of these data sources, including researchers, planners, and policymakers, should factor these into consideration when utilizing the data. Additionally, partners should work with governments to encourage more reliable, comprehensive, and accurate reporting of vaccine and immunization spending.

## CRediT authorship contribution statement

**Gloria Ikilei:** Methodology, Writing - original draft, Writing - review & editing. **Steve D Bachmeier:** Formal analysis, Data curation, Visualization. **Ian E Cogswell:** Formal analysis, Data curation, Visualization. **Emilie R Maddison:** Formal analysis, Data curation, Visualization. **Hayley N Stutzman:** Formal analysis, Data curation, Visualization. **Golsum Tsakalos:** Resources, Supervision, Project administration. **Logan Brenzel:** Writing - review & editing, Funding acquisition. **Joseph L Dieleman:** Conceptualization, Methodology, Writing - review & editing, Supervision, Funding acquisition. **Angela E Micah:** Conceptualization, Methodology, Writing - original draft, Writing - review & editing, Supervision, Funding acquisition.

## Declaration of Competing Interest

The authors declare that they have no known competing financial interests or personal relationships that could have appeared to influence the work reported in this paper.
