# Supplementary material for: Tracking government spending on immunization: The joint reporting forms, national health accounts, comprehensive multi-year plans and co-financing data
Source: Vaccine. 2021 Jun 8;39(25):3410–8. doi: 10.1016/j.vaccine.2021.04.047 (PMC8186488; doi:10.1016/j.vaccine.2021.04.047)
Supplement: Supplementary data 1 [file mmc1.pdf]

Tracking government spending on immunization: The Joint Reporting Forms, National Health Accounts, Comprehensive Multi-Year Plans and Co-financing data

Methods Annex

Institute for Health Metrics and Evaluation

April 2021

Table of Contents

Section 1. Introduction

Section 2. Identifying outliers and excluding data

Section 3. Supplementary tables

Section 1. Introduction

Objective of the study

This study aims to utilize the most recent data to provide an updated comparison of available data sources on government spending on immunization, with a focus on identifying strengths and limitations of the available data.

Section 2. Identifying outliers and excluding data

In the JRF, we found three data points where vaccine spending exceeded routine immunization spending for Dominica in 2011, Uzbekistan in 2014 and Malaysia in 2015. There were no such implausible data points when we compared spending on routine vaccines to total routine spending reported in the CMYP data.

We assessed the reliability of the estimates of government spending on immunization we calculated using data from the NHA by comparing them against overlapping country years of data reported through the GHED. The correlation for country years with overlapping data was 0.99. We prioritized GHED data over data from the NHA for country years with overlapping data.

Under reporting in the JRF, defined as missing data or zero reporting was relatively frequent at 23.5% for routine immunization, Gavi under reporting of more than 50% for both spending categories was noted in thirteen countries including Somalia, Haiti and Libya.

Section 3. Supplementary tables

Cost components of government expenditure on routine immunization by source

**Cost components**	**cMYP/FSP**	**SHA/GHED***	**JRF**
Routine vaccines	Included	Included	Included
Injection supplies	Included	Included	Included
Salaries and per diems of health staff	Included	Included	Included
Transport specific for immunization	Included	Included**	Included
Vehicles and cold-chain maintainence (recurrent costs)	Included	Included**	Included
Immunization-specific training (recurrent costs)	Included	Included	Included
Social mobilization	Included	Excluded***	Included
Monitoring and surveillance	Included	Excluded***	Included
Programme management	Included	Included	Included
Capital costs	Included	Excluded	Excluded
Shared health system costs	Excluded	Included**	Excluded
Mass vaccination campaigns	Excluded	Included	Excluded

*SHA/GHED represents government spending on immunization programs (including supplementary immunization activities)

**Not specified, assumed

***Assumed included in other HC.6 “Preventive care” health functions in SHA/GHED

Table 1.

Data inclusion by country and year

Country	ISO3 code	2000	2001	2002	2003	2004	2005	2006	2007	2008	2009	2010
Afghanistan	AFG		1	1	1			1, 4	4	4	1, 2, 3, 4, 5	3, 4, 5
Albania	ALB		1					4, 5	1, 2, 4, 5	4, 5	4, 5	5
Algeria	DZA										4, 5	
Angola	AGO							4, 5	4, 5	5	5	1, 2, 4, 5
Argentina	ARG							4, 5	4, 5	4, 5	4, 5	4, 5
Armenia	ARM		1				1	5	5	5	1, 2, 3, 4, 5	3, 4, 5
Azerbaijan	AZE		1					5	4, 5	4, 5	1, 2, 4, 5	4, 5
Bangladesh	BGD	6	6	6		1, 6	6	4, 5, 6	4, 5, 6	4, 5, 6	1, 2, 3, 4, 5, 6	3, 4, 5, 6
Belarus	BLR							4, 5	4, 5	4, 5	4, 5	4, 5
Belize	BLZ							4, 5	4, 5	4, 5	4, 5	4, 5
Benin	BEN		1	1, 2	1		1	5	4, 5	1, 3, 4, 5	3, 4, 5	3, 4, 5
Bhutan	BTN		1	1	1			1, 4, 5	4, 5	4	3, 4	3, 4, 5
Bolivia (Plurinational State of)	BOL							4, 5	4, 5	3, 4, 5	3, 4, 5	3, 4, 5
Bosnia and Herzegovina	BIH			1				5	5	5	5	5
Botswana	BWA					1, 2		4	4	4	4, 5	4, 5
Brazil	BRA								4, 5	4, 5	4, 5	4, 5
Bulgaria	BGR							5	5	5	5	4, 5
Burkina Faso	BFA		1, 2	1, 2			1, 2	4, 5	4, 5	4, 5	4, 5	3, 4, 5
Burundi	BDI		1				1	4, 5	4, 5	3, 4, 5	3, 4, 5	1, 2, 3, 4, 5, 6
Cabo Verde	CPV						1, 2	5	5	5	5	5
Cambodia	KHM		1					4	1, 2, 4, 5	4, 5	4, 5	3, 4, 5
Cameroon	CMR		1, 2	1	1		1	4, 5	4, 5	3, 4, 5	3, 4, 5	1, 2, 3, 4, 5
Central African Republic	CAF						1	4	4, 5	3, 4, 5	3, 4, 5	1, 2, 3, 4, 5
Chad	TCD							1, 2, 4, 5	4, 5	3, 4, 5	3, 4, 5	3, 4, 5
China	CHN									4, 5	4, 5	4, 5
Colombia	COL							4, 5	4, 5	4, 5	4, 5	4, 5
Comoros	COM		1	1	1		1	1, 4	4	4	3, 4, 5	3, 4, 5
Congo	COG						1	5	4, 5	3, 4, 5	3, 4, 5	1, 2, 3, 4, 5
Costa Rica	CRI									4, 5	5	4, 5
Cuba	CUB							4, 5	4, 5	4, 5	4, 5	4, 5
Côte d'Ivoire	CIV		1				1	1, 2, 4, 5	4, 5	4, 5	3, 4, 5	3, 4, 5
Democratic People's Republic of Korea	PRK		1	1	1		1		4	4	3, 4, 5	1, 2, 3, 4, 5
Democratic Republic of the Congo	COD			1	1	1	1	1, 4			3, 4, 5	3, 4, 5
Djibouti	DJI						1	4	4	4	4	1, 4, 5

Dominica	DMA										4, 5	4, 5
Dominican Republic	DOM							4, 5	4, 5	4, 5	4, 5	4, 5
Ecuador	ECU							4, 5	4, 5	4, 5	4, 5	4, 5
Egypt	EGY							4, 5	4, 5	5	5	4, 5
El Salvador	SLV							4, 5	4, 5	4, 5	4, 5	4, 5
Equatorial Guinea	GNQ								4, 5	4, 5	4, 5	4, 5
Eritrea	ERI		1	1	1		1					
Eswatini	SWZ							4, 5	4, 5	4, 5	4, 5	4, 5
Ethiopia	ETH		1, 2	1, 2	1, 2		1				1, 2, 4, 5	4, 5
Fiji	FJI							5	5, 6	6	6	5, 6
Gabon	GAB							4, 5	4, 5	4, 5	4, 5	4, 5
Gambia	GMB			1, 2			1	4, 5	4, 5	3, 5	3, 5	1, 2, 3, 5
Georgia	GEO		1					4, 5	4, 5	4, 5	3, 4, 5	1, 2, 3, 4, 5
Ghana	GHA							5	5	3, 4, 5	3	3
Grenada	GRD							4, 5	4, 5	4, 5	4, 5	4, 5
Guatemala	GTM							4, 5	4, 5	4, 5	4, 5	4, 5
Guinea	GIN		1	1	1, 2		1	4, 5	4, 5	3, 4, 5	3, 4, 5	1, 3, 4, 5
Guinea-Bissau	GNB					1				3	3	3
Guyana	GUY	1						4, 5	4, 5	4, 5	3, 4, 5	3, 4, 5
Haiti	HTI		1					1, 4	4			
Honduras	HND							4, 5	4, 5	3, 4, 5	3, 4, 5	3, 4, 5
India	IND							4, 5	4, 5	4, 5	4, 5	4, 5
Indonesia	IDN							4, 5	4, 5	4, 5	1, 2, 4, 5	4, 5
Iran (Islamic Republic of)	IRN							5	5	5	5	4, 5
Iraq	IRQ							4, 5	4, 5	4, 5	4	
Jamaica	JAM							4, 5	4, 5	4, 5	4, 5	4, 5
Jordan	JOR							4, 5	4, 5	4, 5	4, 5	4, 5
Kazakhstan	KAZ							5	4, 5	4, 5	5	4, 5
Kenya	KEN	1					1	4, 5	4, 5	3, 4, 5	3, 4, 5	1, 2, 3, 4, 5
Kiribati	KIR								5	3, 5	3	1, 2, 3
Kyrgyzstan	KGZ		1				1	4, 5	5	5	1, 2, 3, 4, 5	3, 4, 5
Lao People's Democratic Republic	LAO	1						1, 2, 4	4, 5	4, 5	3, 4, 5	3, 4, 5
Lebanon	LBN							4, 5	4, 5	4, 5	4, 5	4, 5
Lesotho	LSO		1	1	1		1			3	3	1, 2, 3
Liberia	LBR						1	4	4, 5	3, 4, 5, 6	1, 2, 3, 5	3, 5
Libya	LBY								5	4, 5	4, 5	
Madagascar	MDG	1, 2	1, 2	1, 2	1, 2		1, 2	4, 5	4, 5	3, 4, 5	3, 4, 5	1, 2, 3, 4, 5, 6
Malawi	MWI	1	1	1	6	6	1, 6	5	4, 5, 6	1, 2, 3, 5, 6	3, 4, 5, 6	3, 4, 5
Malaysia	MYS							5	4, 5	4, 5	4, 5	4, 5
Maldives	MDV							4, 5	4, 5			4, 5
Mali	MLI	1					1	4, 5	4, 5	3, 5	1, 2, 3, 4, 5	3, 4, 5
Marshall Islands	MHL							4, 5	4, 5	5	4, 5	4, 5

Mauritania	MRT		1					5	4, 5	4, 5	3, 4, 5	1, 2, 3, 4, 5
Mauritius	MUS							5	5	5	5	5
Mexico	MEX							4	4, 5	4, 5	4, 5	4, 5
Micronesia (Federated States of)	FSM							4, 5	4, 5	4, 5	4, 5	4, 5
Mongolia	MNG					1, 2	1	5	5	5	5	3, 5
Montenegro	MNE											
Morocco	MAR							4, 5	4, 5	4, 5	4, 5	5
Mozambique	MOZ	1					1	5	4, 5	4, 5	3, 4, 5	1, 2, 3, 4, 5
Myanmar	MMR		1			1						4
Namibia	NAM							5	4, 5	5	5	4, 5
Nepal	NPL		1, 2	1, 2	1, 2		1	4, 5	4, 5	4, 5	3, 4, 5	1, 2, 3, 4, 5, 6
Nicaragua	NIC							4, 5	4, 5	4, 5	3, 4, 5	3, 4, 5
Niger	NER		1, 2	1, 2	1, 2		1	5	5	3, 5	1, 2, 3, 5	3, 4, 5
Nigeria	NGA						1, 2	4, 5	5	1, 2, 5	5	4, 5
North Macedonia	MKD							5	4, 5	4, 5	4, 5	5
Pakistan	PAK						1	4, 5	4, 5	1, 2, 3, 4, 5	3, 4, 5	3, 4, 5
Papua New Guinea	PNG							4, 5	4, 5	4, 5	3, 4, 5	3, 4, 5
Paraguay	PRY							4, 5	4, 5	4, 5	4, 5	4, 5
Peru	PER							4, 5	4, 5	4, 5	4, 5	4, 5
Philippines	PHL							4, 5	4, 5	4, 5	4, 5	4, 5
Republic of Moldova	MDA		1, 2	1, 2	1, 2			5	5	3, 5	1, 2, 3, 4, 5	3, 4, 5
Romania	ROU								5	4, 5	4, 5	5
Russian Federation	RUS											
Rwanda	RWA	1	1				1	4, 5	4, 5	3, 4, 5	3, 4, 5	1, 2, 3, 4, 5
Saint Lucia	LCA								4, 5	4, 5	4, 5	4, 5
Saint Vincent and the Grenadines	VCT								4, 5	4, 5	4, 5	4, 5
Samoa	WSM							4, 5	4, 5	4, 5	4, 5	4, 5
Sao Tome and Principe	STP								4, 5	4, 5	3, 4, 5	1, 2, 3, 4, 5
Senegal	SEN		1				1	5	4, 5	4, 5	3, 5	1, 2, 3, 4, 5
Sierra Leone	SLE		1	1	1		1, 2			3, 4, 5	3, 5	3
Solomon Islands	SLB							4	4	3, 4, 5	3, 5	3, 4, 5
Somalia	SOM											1
South Africa	ZAF										4, 5	4, 5
South Sudan	SSD											
Sri Lanka	LKA		1, 2	1, 2	1, 2			1, 2, 4, 5	4, 5	4, 5	4, 5	3, 4, 5
Sudan	SDN		1	1	1		1	4	4	3, 4	3, 4, 5	1, 2, 3, 4, 5
Suriname	SUR							4, 5	4, 5	4, 5	4, 5	4, 5
Syrian Arab Republic	SYR							4, 5	5	5	4, 5	5
Tajikistan	TJK		1	1			1	5	4, 5	3, 4, 5	1, 2, 3, 4, 5	3, 4, 5
Thailand	THA							5	5	4, 5	4, 5	4, 5
Timor-Leste	TLS							4			5	1, 2, 4, 5
Togo	TGO						1	5	4, 5	3, 4, 5	1, 2, 3, 4, 5	3, 4, 5

Tonga	TON							5	4, 5	4, 5	4, 5	4, 5
Tunisia	TUN							4, 5	4, 5	4, 5	4, 5	4, 5
Turkey	TUR									4, 5	4, 5	4, 5
Turkmenistan	TKM							5	5	5	5	5
Uganda	UGA		1, 2	1, 2		1		4, 5	4, 5	1, 2, 3, 4, 5	3, 4, 5	3, 4, 5
Ukraine	UKR		1					4, 5	4, 5	4, 5	4, 5	4, 5
United Republic of Tanzania	TZA		1	1			1	4, 5	4, 5	3, 4, 5	3, 4, 5	1, 2, 3, 4, 5
Uzbekistan	UZB	1, 2		1, 2				4, 5	4, 5	4, 5	1, 2, 3, 4, 5	3, 4, 5
Vanuatu	VUT							4, 5	4, 5	4, 5	4, 5	4
Venezuela (Bolivarian Republic of)	VEN										4, 5	4, 5
Viet Nam	VNM	1						4, 5	4, 5	4, 5	4, 5	3, 4, 5
Yemen	YEM		1, 2	1, 2	1, 2		1	4, 5	4, 5	3, 4, 5	1, 2, 3, 4, 5	3, 4, 5
Zambia	ZMB	1						4, 5	4, 5	3, 5	3, 5	1, 2, 3, 4, 5
Zimbabwe	ZWE		1, 2	1, 2	1, 2		1					1, 4
		14	50	42	30	11	46	183	204	242	297	330

Country	ISO3 code	2011	2012	2013	2014	2015	2016	2017	Total
Afghanistan	AFG	3, 4, 5	3, 4, 5	1, 2, 3, 4, 5	3, 4, 5, 6	3, 4, 5	3, 4, 5, 6	3, 4, 5, 6	41
Albania	ALB					5	4, 5	5	16
Algeria	DZA		5	5	4, 5	4, 5	4, 5	4, 5	12
Angola	AGO	3, 5	3, 5	3, 5	3, 5	3, 5	3, 5	3, 5	24
Argentina	ARG	4, 5	4, 5	4, 5	4, 5	4, 5	4, 5	4, 5	24
Armenia	ARM	3, 4, 5	3, 4, 5	3, 4, 5	1, 2, 3, 4, 5, 6	3, 4, 5, 6	3, 4, 5, 6	3, 4, 5	39
Azerbaijan	AZE	3, 4, 5	3, 4, 5	3, 4, 5	1, 2, 3, 4, 5	3, 4, 5	3, 4, 5	3, 4, 5	35
Bangladesh	BGD	3, 4, 5, 6	3, 4, 5, 6	3, 4, 5	3, 4, 5	3, 4, 5	3, 4, 5	3, 4, 5	48
Belarus	BLR	4, 5	4, 5	4, 5	4, 5	4, 5	4, 5	4, 5	24
Belize	BLZ	4, 5	4, 5	4, 5	4, 5	4, 5	4, 5	4, 5	24
Benin	BEN	3, 4, 5	3, 4, 5	1, 2, 3, 4, 5	3, 4, 5	3, 4, 5	3, 4, 5, 6	3, 4, 5, 6	43
Bhutan	BTN	3, 4, 5	1, 2, 3, 5	3, 5	3, 4, 5	3, 4, 5, 6	4, 5, 6	4, 5, 6	36
Bolivia (Plurinational State of)	BOL	3, 4, 5	3, 4, 5	3, 4, 5	3, 4, 5	3, 4, 5	3, 4, 5	3, 4, 5	34
Bosnia and Herzegovina	BIH	5	5	5	5	5	4, 5, 6		14
Botswana	BWA	4, 5	4, 5	4, 5	4, 5	4, 5	4, 5, 6	4, 5, 6	25
Brazil	BRA	4, 5	4, 5	4, 5	4, 5	4, 5	4, 5	4, 5	22
Bulgaria	BGR	4, 5	4, 5	4, 5	4, 5	4, 5	4, 5	4, 5	20
Burkina Faso	BFA	3, 4, 5	3, 4, 5	3, 4, 5	3, 4, 5	3, 4, 5	3, 4, 5, 6	3, 4, 5, 6	40
Burundi	BDI	3, 4, 5	3, 4, 5, 6	3, 4, 5, 6	3, 4, 5	3, 4, 5	3, 4, 5, 6	3, 4, 5, 6	43
Cabo Verde	CPV	5	5	5	5	5	5	5	14
Cambodia	KHM	3, 4, 5	3, 4, 5	3, 4, 5	3, 4, 5	3, 4, 5	3, 4, 5, 6	3, 4, 5, 6	36
Cameroon	CMR	3, 4, 5	3, 4, 5, 6	1, 2, 3, 4, 5	3, 4, 5	3, 4, 5	3, 4, 5	3, 4, 5	44
Central African Republic	CAF	3, 4, 5	3, 4, 5	4, 5	1, 2, 3, 4, 5	3, 4, 5	3, 4, 5, 6	3, 4, 5, 6	39
Chad	TCD	3, 4, 5	3, 4, 5	1, 2, 3, 4, 5	3, 4, 5	3, 4, 5	3, 4, 5	3, 4, 5	38
China	CHN	4, 5	4, 5	4, 5	4, 5	4, 5	4, 5	4, 5	20

Colombia	COL	4, 5	4, 5	4, 5	4, 5	4, 5	4, 5	4, 5	24
Comoros	COM	3, 4, 5	3, 4, 5	3, 4, 5	3, 4, 5	1, 2, 3, 4, 5	3, 4, 5	3, 4, 5	37
Congo	COG	3, 4, 5	3, 4, 5	3, 4, 5	3, 4, 5	3, 4, 5	3, 4, 5, 6	3, 4, 5, 6	38
Costa Rica	CRI	4, 5, 6	4, 5, 6	4, 5, 6	4, 5, 6	4, 5, 6	4, 5, 6	4, 5, 6	26
Cuba	CUB	4, 5	4, 5	4, 5	4, 5	4, 5	4, 5	4, 5	24
Côte d'Ivoire	CIV	3, 4, 5	3, 4, 5	3, 4, 5	3, 4, 5	1, 2, 3, 4, 5, 6	3, 4, 5, 6	3, 4, 5, 6	42
Democratic People's Republic of Korea	PRK	3, 4, 5	3, 4, 5	3, 4, 5	3, 4, 5	3, 4, 5	3, 4, 5	3, 4, 5	35
Democratic Republic of the Congo	COD	3, 4, 5	3, 4, 5	1, 2, 3, 4, 5, 6	3, 4, 5, 6	3, 4, 5, 6	3, 4, 5, 6	4, 6	38
Djibouti	DJI	4	3, 4	3, 4, 5	3, 4, 5	3, 4, 5	3, 4, 5	3, 4, 5	26
Dominica	DMA		4, 5	4, 5	4, 5	4, 5	4, 5	4, 5	16
Dominican Republic	DOM	4, 5	4, 5	4, 5	4, 5, 6	4, 5	4, 5, 6	4, 5, 6	27
Ecuador	ECU	4, 5	4, 5	4, 5	4, 5	4, 5	4, 5	4, 5	24
Egypt	EGY	4, 5	4, 5	4, 5	4, 5	4, 5	4, 5	4, 5	22
El Salvador	SLV	4, 5	4, 5	4, 5	4, 5	4, 5	4, 5	4, 5	24
Equatorial Guinea	GNQ	4, 5	4, 5	4, 5	4, 5	1, 2, 4, 5	4, 5	4, 5	24
Eritrea	ERI	3, 4, 5	3, 4, 5	3, 4, 5	3, 4, 5	3, 4, 5	3, 4, 5	3, 4, 5	25
Eswatini	SWZ	4, 5	4, 5	4, 5	4, 5	4, 5	4, 5	4, 5, 6	25
Ethiopia	ETH	3, 4, 5	3, 4, 5	3, 4, 5	1, 2, 3, 4, 5	3, 4, 5	3, 4, 5, 6	3, 4, 5, 6	38
Fiji	FJI	5, 6	5, 6	5, 6	5, 6	4, 5, 6	5, 6	5, 6	22
Gabon	GAB	4, 5	4, 5	4, 5	4, 5	4, 5	4, 5, 6	4, 5, 6	26
Gambia	GMB	3, 5	3, 4, 5	3, 4, 5	3, 4, 5	1, 2, 3, 4, 5	3, 4, 5	3, 4, 5	37
Georgia	GEO	3, 4, 5	3, 4, 5	3, 4, 5	3, 4, 5	3, 4, 5	3, 4, 5, 6	3, 4, 5, 6	38
Ghana	GHA	3	3, 4, 5	3, 4, 5	1, 3, 4, 5	3, 4, 5	3, 4, 5, 6	3, 4, 5, 6	29
Grenada	GRD	4, 5	4, 5	4, 5	4, 5	4, 5	4, 5	4, 5	24
Guatemala	GTM	4, 5	4, 5	4, 5	4, 5	4, 5	4, 5	4, 5	24
Guinea	GIN	3, 4, 5	3, 4, 5	3, 4, 5	4, 5	4, 5	3, 4, 5, 6	3, 4, 5, 6	40
Guinea-Bissau	GNB	3, 4, 5	3, 5	3, 5	3, 5	3, 4, 5	3, 4, 5	3, 4, 5	22
Guyana	GUY	3, 4, 5	3, 4, 5	3, 4, 5	3, 4, 5	3, 4, 5	3, 4, 5, 6	3, 4, 5	35
Haiti	HTI		3		3	3	3	3, 4	10
Honduras	HND	3, 4, 5	3, 4, 5	3, 4, 5	3, 4, 5	3, 4, 5	4, 5	4, 5	32
India	IND	4, 5	1, 2, 4, 5	4, 5	4, 5, 6	4, 5, 6	4, 5, 6	4, 5	29
Indonesia	IDN	4, 5	4, 5	1, 2, 3, 4, 5	3, 4, 5	3, 4, 5	3, 4, 5	4, 5	32
Iran (Islamic Republic of)	IRN	4, 5	4, 5	4, 5	4, 5	4, 5	4, 5	4, 5	20
Iraq	IRQ			4, 5	4, 5	4, 5	4, 5		15
Jamaica	JAM	4, 5	4, 5	4, 5	4, 5	4, 5	4, 5	4, 5	24
Jordan	JOR	4, 5	4, 5	4, 5	4, 5	4, 5	4, 5	4, 5	24
Kazakhstan	KAZ	4, 5	4, 5	4, 5	4, 5, 6	4, 5	4, 5, 6	4, 5, 6	25
Kenya	KEN	3, 4, 5	3, 4, 5	3, 4, 5	1, 2, 3, 4, 5	3, 4, 5	3, 4, 5	3, 5	39
Kiribati	KIR	3, 4, 5	3	3	3	3, 5	3, 5	4, 5	19
Kyrgyzstan	KGZ	3, 4, 5	3, 4, 5	3, 4, 5	3, 4, 5	3, 4, 5	3, 4, 5, 6	3, 4, 5, 6	37
Lao People's Democratic Republic	LAO	3, 4, 5	3, 4, 5	3, 4, 5	3, 4, 5	3, 4, 5	3, 4, 5, 6	1, 2, 3, 4, 5, 6	39
Lebanon	LBN	4, 5	4, 5	4, 5	4, 5	4, 5	4, 5	4, 5	24

Lesotho	LSO	3, 4, 5	3, 4, 5	3, 4, 5	3, 4, 5	3, 4, 5	3, 4, 5	3, 4, 5	30
Liberia	LBR	3, 5	3, 5	3, 4, 5	1, 2, 3, 4, 5	4	3, 4, 5	3, 4, 5	33
Libya	LBY								5
Madagascar	MDG	3, 4, 5	3, 4, 5	3, 4, 5	1, 2, 3, 4, 5	3, 4, 5	3, 4, 5	3, 4, 5	49
Malawi	MWI	3, 4, 5	3, 5	3, 4, 5, 6	1, 2, 3, 4, 5, 6	3, 4, 5, 6	3, 4, 5, 6	3, 4, 5, 6	50
Malaysia	MYS	4, 5	5	5	5		4, 5	5	17
Maldives	MDV	4, 5	4, 5	4, 5	4, 5	4, 5	4, 5	4, 5	20
Mali	MLI	3, 4, 5	3, 4, 5	3, 4, 5, 6	3, 4, 5, 6	3, 4, 5, 6	3, 4, 5, 6	3, 4, 5, 6	42
Marshall Islands	MHL	4, 5	4, 5	4, 5	4, 5	4, 5	4, 5	4, 5	23
Mauritania	MRT	3, 4, 5	3, 4, 5	3, 4, 5	1, 2, 3, 4, 5	3, 4, 5, 6	3, 4, 5, 6	3, 4, 5, 6	40
Mauritius	MUS	4, 5	4, 5	4, 5	4, 5	4, 5, 6	4, 5, 6	5	20
Mexico	MEX	4, 5			4, 5	4, 5	4, 5	4, 5	19
Micronesia (Federated States of)	FSM								10
Mongolia	MNG	3, 4, 5	3, 4, 5	3, 4, 5	3, 4, 5	3, 4, 5	4, 5	4, 5	28
Montenegro	MNE						5	5	2
Morocco	MAR	5	5	5	5	5	5	5	16
Mozambique	MOZ	3, 4, 5	3, 4, 5	3, 4, 5	3, 4, 5	3, 4, 5	3, 4, 5	3, 4, 5	36
Myanmar	MMR	1, 4	3, 4, 5	3, 4, 5	3, 4, 5	1, 2, 3, 4, 5	3, 4, 5, 6	3, 5, 6	26
Namibia	NAM	4, 5	4, 5	5			4, 5, 6	4, 5, 6	18
Nepal	NPL	3, 4, 5, 6	3, 4, 5, 6	3, 4, 5, 6	3, 4, 5, 6	3, 4, 5, 6	3, 4, 5, 6	3, 4, 5, 6	50
Nicaragua	NIC	3, 4, 5	3, 4, 5	3, 4, 5	3, 4, 5	3, 4, 5	3, 4, 5	3, 4, 5	33
Niger	NER	3, 5, 6	3	3, 5, 6	3, 5, 6	3, 4, 5, 6	3, 4, 5, 6	3, 4, 5, 6	40
Nigeria	NGA	3	3	1, 2, 3, 5	3, 4, 5	3, 4, 5	3, 4, 5, 6	3, 4, 5, 6	31
North Macedonia	MKD	5	4, 5	4, 5	5	5	4, 5	5	18
Pakistan	PAK	3, 4, 5	1, 3, 4, 5	3, 4, 5	3, 4, 5	3, 4, 5	3, 4, 5, 6	3, 4, 5	39
Papua New Guinea	PNG	3, 4, 5	3, 4, 5	3, 4, 5	3, 4, 5	3, 4, 5	3, 4, 5	3, 4, 5	33
Paraguay	PRY	4, 5	4, 5	4, 5	4, 5, 6	4, 5	4, 5	4, 5, 6	26
Peru	PER	4, 5	4, 5	4, 5	4, 5	4, 5	4, 5	4, 5	24
Philippines	PHL	5	5	4, 5	4, 5	4, 5	4, 5	4, 5	22
Republic of Moldova	MDA	3, 5	3, 5	3, 4, 5	1, 2, 3, 4, 5	3, 4, 5	3, 4, 5	4, 5	38
Romania	ROU	5	5	5	5	5	5	5	13
Russian Federation	RUS					5	5, 6	4, 5, 6	6
Rwanda	RWA	3, 4, 5	3, 4, 5	3, 4, 5	3, 4, 5	3, 4, 5	1, 2, 3, 4, 5	3, 4, 5	41
Saint Lucia	LCA	4, 5	4, 5	4, 5	4, 5	4, 5	4, 5	4, 5	22
Saint Vincent and the Grenadines	VCT	4, 5	4, 5	4, 5	4, 5	4, 5	4, 5	4, 5	22
Samoa	WSM	4, 5	4, 5			6		5	16
Sao Tome and Principe	STP	3, 4, 5	3, 4, 5	3, 4, 5	1, 2, 3, 4, 5	3, 4, 5	3, 4, 5	3, 4, 5	35
Senegal	SEN	3, 4, 5	3, 4, 5	1, 2, 3, 4, 5	3, 4, 5	3, 4, 5, 6	3, 4, 5, 6	3, 4, 5, 6	40
Sierra Leone	SLE	3, 4, 5	3, 4, 5	3, 4, 5	4, 5	4	3, 4, 5	3, 4, 5	29
Solomon Islands	SLB	3, 4, 5	3, 4, 5	3, 4, 5	3, 4, 5	3, 4, 5	3, 4, 5	3, 4, 5	31
Somalia	SOM			3	3	3, 5	3	3	7
South Africa	ZAF	5	5	5	5, 6	5	5	4, 5	13

South Sudan	SSD	1, 4			3, 5	4, 5	4, 5	4, 5, 6	11
Sri Lanka	LKA	1, 2, 3, 4, 5	3, 4, 5	3, 4, 5	3, 4, 5	3, 4, 5	4, 5, 6	3, 5	41
Sudan	SDN	3, 4, 5	3, 4, 5	3, 4, 5	3, 4, 5	3, 4, 5	3, 4, 5	3, 4, 5	37
Suriname	SUR	4, 5	5	5	4, 5	4, 5	4, 5	4, 5	22
Syrian Arab Republic	SYR	5	5	5	5	5	4, 5	4, 5	16
Tajikistan	TJK	3, 4, 5	3, 4, 5	3, 4, 5	1, 2, 3, 4, 5	3, 4, 5	3, 4, 5, 6	3, 4, 5, 6	42
Thailand	THA	4, 5	4, 5	4, 5	4, 5	4, 5	4, 5	5	21
Timor-Leste	TLS	4, 5	3, 4, 5	3, 4, 5	3, 4, 5	3, 4, 5	3, 4, 5	3, 4, 5	26
Togo	TGO	3, 4, 5	3, 4, 5	3, 4, 5	1, 2, 3, 4, 5	3, 4, 5	3, 4, 5, 6	3, 4, 5, 6	40
Tonga	TON	4, 5	4, 5	4, 5	4, 5	4, 5	4, 5	4, 5	23
Tunisia	TUN	4, 5	4, 5	4, 5	4, 5	4, 5	4, 5, 6	4, 5	25
Turkey	TUR	4, 5	4, 5	4, 5	4, 5	4, 5	4, 5		18
Turkmenistan	TKM	5	5	5	4, 5	4, 5	4, 5	4, 5	16
Uganda	UGA	3, 5, 6	3, 5, 6	3, 4, 5, 6	1, 2, 3, 4, 5, 6	3, 4, 5, 6	3, 4, 5, 6	3, 4, 5, 6	48
Ukraine	UKR	4, 5					4, 5	4, 5	17
United Republic of Tanzania	TZA	1, 2, 3, 4, 5	3, 4, 5	3, 4, 5	3, 4, 5	3, 4, 5	3, 4, 5, 6	3, 5, 6	42
Uzbekistan	UZB	3, 4, 5	3, 4, 5	3, 4, 5	1, 2, 3	3, 4, 5	3, 4, 5, 6	3, 4, 5, 6	41
Vanuatu	VUT	4, 5	4, 5	4, 5	4, 5	4, 5	4, 5	4, 5	23
Venezuela (Bolivarian Republic of)	VEN	4, 5	4, 5	4, 5	4, 5	4, 5	4, 5		16
Viet Nam	VNM	3, 4, 5	3, 4, 5	3, 4, 5	1, 2, 3, 4, 5	3, 4, 5	3, 4, 5	3, 4, 5	35
Yemen	YEM	3, 4, 5	3, 4, 5	3, 4, 5	3, 4, 5	4, 5	4, 5	3	39
Zambia	ZMB	3, 4, 5	3, 4, 5	3, 4, 5	3, 4, 5	3, 4, 5	3, 4, 5, 6	3, 4, 5, 6	37
Zimbabwe	ZWE	4	3, 4	3, 4, 5	3, 4, 5	3, 4, 5	3, 4, 5	3, 4, 5	27
		294	294	315	343	325	357	335	3712

Note: 1 = cMYP/FSP: Government spending on routine immunization. 2 = cMYP/FSP: Government spending on vaccines. 3 = Gavi co-financing of vaccines. 4 = JRF: Government spending on routine immunization. 5 = Government spending on vaccines. 6 = SHA/GHED: Government spending on immunization programs.

Table 2.

Gavi co-financing of vaccines to JRF Government spending on vaccines ratio by country and year

Country	ISO3 code	2008	2009	2010	2011	2012	2013	2014	2015	2016	2017
Afghanistan	AFG		1	1	0.65	0.56	1.23	0.92	0.84	0.93	1.27
Angola	AGO				0.16	0.31	0.32	0.53	0.72	0.43	0.11
Armenia	ARM		0.1	0.16	0.22	0.2	0.32	0.43	0.17	0.16	0.25
Azerbaijan	AZE				0.27	0.21	0.42	0.89	0.49	0.32	0.36
Bangladesh	BGD		0.2	0.14	0.14	0.2	0.26	0.14	0.19	0.19	0.22
Benin	BEN	0.45	0.13	0.33	0.35	0.53	0.38	0.35	0.3	0.32	0.25
Bhutan	BTN			1	1.01	0.97	1.05	0.91	1.11		
Bolivia (Plurinational State of)	BOL	0.14	0.21	0.2	0.24	0.1	0.07	0.19	0.14	0.12	0.24
Burkina Faso	BFA			0.18	0.05	0.14	0.29	0.45	0.73	0.28	0.24

Country	ISO3 code	2008	2009	2010	2011	2012	2013	2014	2015	2016	2017
Burundi	BDI	1	1	0.58	0.81	0.6	1	0.99	0.99	1	0.49
Cambodia	KHM			0.31	0.27	0.26	0.17	0.08	0.28	0.26	0.13
Cameroon	CMR	0.14	0.32	0.29	0.68	0.73	0.63	0.76	0.76	0.57	0.38
Central African Republic	CAF	0.98	2.68	0.84	4.28	3.38		0.96	1	1.48	1.55
Chad	TCD	0.13	0.16	0.16	0.3	0.22	0.16	0.19	0.34	0.33	0.56
Comoros	COM		0.96	0.78	0.97	0.56	0.71	0.68	1	0.66	0.47
Congo	COG	0.1	0.36	0.12	0.49	1.41	2.39	1.56	1.56	0.47	1.78
Côte d'Ivoire	CIV		0.05	0.03	0.08	0.55	0.06	0.37	0.24	0.18	0.11
Democratic People's Republic of Korea	PRK		1	0.46	1.02	0.52	0.93	1.36	0.59	0.72	0.9
Democratic Republic of the Congo	COD		1.06	0.76	3.24	0.64	0.54	2.5	3.35	2.66	
Djibouti	DJI						0.86	0.99	0.99	0.96	1.26
Eritrea	ERI				7.32	1.02	1	1.08	1	0.88	0.9
Ethiopia	ETH				0.18	0.56	0.71	0.7	0.46	0.37	0.37
Gambia	GMB	0.11	0.3	0.54	0.41	0.38	0.42	0.3	0.19	0.16	0.13
Georgia	GEO		0.04	0.05	0.16	0.28	0.13	0.51	0.12	0.07	0.08
Ghana	GHA	0.08				0.64	0.7	0.89	0.46	0.3	0.64
Guinea	GIN	0.09	0.47	0.96	0.75	2.52	0.88			0.68	1.2
Guinea-Bissau	GNB				1	0.99	1	1.44	1	1.01	0.54
Guyana	GUY		0.01	0.01	0.03	0.05	0.13	0.15	0.3	0.2	0.11
Honduras	HND	0.01	0	0.02	0.03	0.22	0.2	0.3	0.29		
Indonesia	IDN						0.04	0.31	0.27	0.23	
Kenya	KEN	0.68	0.52	0.53	0.51	0.71	0.64	0.44	0.94	0.2	0.26
Kiribati	KIR	0.19			0.51				0.62	0.26	
Kyrgyzstan	KGZ		0.43	0.22	0.21	0.18	0.17	0.28	0.17	0.16	0.29
Lao People's Democratic Republic	LAO		0.56	1.04	1.08	0.61	0.56	0.6	0.07	0.21	0.36
Lesotho	LSO				0.21	0.14	0.08	0.97	0.63	0.07	0.22
Liberia	LBR	0.67	1.21	0.99	1.06	1.05	1.19	1		1	1.08
Madagascar	MDG	0.91	0.71	1.81	0.33	1.13	0.61	0.97	0.73	0.94	0.43
Malawi	MWI	0.18	0.21	0.16	0.2	0.63	1.1	0.79	0.9	1.32	1.1
Mali	MLI	0.05	0.06	0.03	0.17	0.53	0.22	0.21	0.24	0.53	0.41
Mauritania	MRT		0.39	0.46	0.27	0.63	0.34	0.45	0.67	0.51	0.36
Mongolia	MNG			0.46	0.42	0.1	0.11	0.21	0.17		
Mozambique	MOZ		0.16	0.24	0.13	0.22	0.33	0.25	0.31	0.37	0.25
Myanmar	MMR					0.4	0.9	1.07	0.85	0.64	0.09
Nepal	NPL		0.29	0.12	0.14	0.18	0.22	0.31	0.26	0.1	0.17
Nicaragua	NIC		0	0.01	0.1	0.08	0.04	0.05	0.09	0.15	0.17
Niger	NER	0.6	0.55	0.74	0.6		0.26	0.99	0.42	0.37	0.35
Nigeria	NGA						0.44	0.23	0.14	0.24	1.07
Pakistan	PAK	0.11	0.18	0.14	0.17	0.81	0.61	0.68	0.33	0.34	0.71
Papua New Guinea	PNG		0.18	0.65	0.5	0.26	0.16	0.47	2.1	0.88	4.3
Republic of Moldova	MDA	0.09	0.09	0.08	0.05	0.17	0.34	0.3	0.63	0.28	

Country	ISO3 code	2008	2009	2010	2011	2012	2013	2014	2015	2016	2017
Rwanda	RWA	0.41	0.98	0.77	0.51	0.57	0.26	0.35	0.52	0.59	0.51
Sao Tome and Principe	STP		0.23	1.39	0.75	0.89	1.65	0.97	1.7	1.39	0.53
Senegal	SEN		0.23	0.19	0.18	0.34	0.25	0.44	0.6	0.7	0.28
Sierra Leone	SLE	1	0.99		1	1	0.86			0.91	0.29
Solomon Islands	SLB	0.08	0.15	0.08	0.9	0.51	0.19	0.44	0.35	0.38	0.44
Somalia	SOM								0.03		
South Sudan	SSD							0.9			
Sri Lanka	LKA			0.12	0.25	0.59	0.3	0.53	0.62		0.21
Sudan	SDN		0.86	0.47	0.64	0.96	0.92	1.22	0.99	1.04	1.07
Tajikistan	TJK	0.97	0.24	0.35	0.43	0.56	0.38	0.42	0.42	0.31	0.39
Timor-Leste	TLS					0.35	0.24	0.16	0.6	0.42	0.44
Togo	TGO	0.25	0.44	0.78	0.2	0.29	0.18	0.6	1.04	1.01	0.79
Uganda	UGA	0.23	0.62	0.39	0.22	0.41	0.56	0.32	0.51	0.46	0.33
United Republic of Tanzania	TZA	0.66	0.19	0.42	0.16	0.27	0.62	0.89	0.51	0.29	0.41
Uzbekistan	UZB		0.24	0.21	0.12	0.21	0.18		0.44	0.63	0.63
Viet Nam	VNM			0.27	0.26	0.31	0.36	0.38	0.29	0.42	0.32
Yemen	YEM	1.16	0.5	0.72	0.67	0.46	0.69	0.65			
Zambia	ZMB	1.14	0.78	0.54	0.21	0.25	0.46	0.77	0.42	0.88	0.82
Zimbabwe	ZWE						1.03	0.97	0.94	1.02	0.73

Table 3.

List of countries included in analysis

Country	Income Classification
Afghanistan	Low-income
Albania	Upper-middle-income
Algeria	Upper-middle-income
American Samoa	Upper-middle-income
Angola	Lower-middle-income
Armenia	Upper-middle-income
Azerbaijan	Upper-middle-income
Bangladesh	Lower-middle-income
Belarus	Upper-middle-income
Belize	Upper-middle-income
Benin	Low-income
Bhutan	Lower-middle-income
Bolivia	Lower-middle-income
Bosnia and Herzegovina	Upper-middle-income
Botswana	Upper-middle-income

Brazil	Upper-middle-income
Bulgaria	Upper-middle-income
Burkina Faso	Low-income
Burundi	Low-income
Cabo Verde	Lower-middle-income
Cambodia	Lower-middle-income
Cameroon	Lower-middle-income
Central African Republic	Low-income
Chad	Low-income
China	Upper-middle-income
Colombia	Upper-middle-income
Comoros	Low-income
Congo, Dem. Rep.	Low-income
Congo, Rep.	Lower-middle-income
Costa Rica	Upper-middle-income
Cuba	Upper-middle-income
Côte d'Ivoire	Lower-middle-income
Djibouti	Lower-middle-income
Dominica	Upper-middle-income
Dominican Republic	Upper-middle-income
Ecuador	Upper-middle-income
Egypt, Arab Rep.	Lower-middle-income
El Salvador	Lower-middle-income
Equatorial Guinea	Upper-middle-income
Eritrea	Low-income
Ethiopia	Low-income
Fiji	Upper-middle-income
Gabon	Upper-middle-income
Gambia, The	Low-income
Georgia	Lower-middle-income
Ghana	Lower-middle-income
Grenada	Upper-middle-income
Guatemala	Upper-middle-income
Guinea	Low-income
Guinea-Bissau	Low-income
Guyana	Upper-middle-income
Haiti	Low-income
Honduras	Lower-middle-income
India	Lower-middle-income
Indonesia	Lower-middle-income
Iran, Islamic Rep.	Upper-middle-income
Iraq	Upper-middle-income

Jamaica	Upper-middle-income
Jordan	Upper-middle-income
Kazakhstan	Upper-middle-income
Kenya	Lower-middle-income
Kiribati	Lower-middle-income
Korea, Dem. Rep.	Low-income
Kosovo	Lower-middle-income
Kyrgyz Republic	Lower-middle-income
Lao PDR	Lower-middle-income
Lebanon	Upper-middle-income
Lesotho	Lower-middle-income
Liberia	Low-income
Libya	Upper-middle-income
Macedonia, FYR	Upper-middle-income
Madagascar	Low-income
Malawi	Low-income
Malaysia	Upper-middle-income
Maldives	Upper-middle-income
Mali	Low-income
Marshall Islands	Upper-middle-income
Mauritania	Lower-middle-income
Mauritius	Upper-middle-income
Mexico	Upper-middle-income
Micronesia, Fed. Sts.	Lower-middle-income
Moldova	Lower-middle-income
Mongolia	Lower-middle-income
Montenegro	Upper-middle-income
Morocco	Lower-middle-income
Mozambique	Low-income
Myanmar	Lower-middle-income
Namibia	Upper-middle-income
Nepal	Low-income
Nicaragua	Lower-middle-income
Niger	Low-income
Nigeria	Lower-middle-income
Pakistan	Lower-middle-income
Papua New Guinea	Lower-middle-income
Paraguay	Upper-middle-income
Peru	Upper-middle-income
Philippines	Lower-middle-income
Romania	Upper-middle-income
Russian Federation	Upper-middle-income

Rwanda	Low-income
Samoa	Upper-middle-income
Senegal	Low-income
Serbia	Upper-middle-income
Sierra Leone	Low-income
Solomon Islands	Lower-middle-income
Somalia	Low-income
South Africa	Upper-middle-income
South Sudan	Low-income
Sri Lanka	Lower-middle-income
St. Lucia	Upper-middle-income
St. Vincent and the Grenadines	Upper-middle-income
Sudan	Lower-middle-income
Suriname	Upper-middle-income
Swaziland	Lower-middle-income
Syrian Arab Republic	Low-income
Sao Tome and Principe	Lower-middle-income
Tajikistan	Low-income
Tanzania	Low-income
Thailand	Upper-middle-income
Timor-Leste	Lower-middle-income
Togo	Low-income
Tonga	Upper-middle-income
Tunisia	Lower-middle-income
Turkey	Upper-middle-income
Turkmenistan	Upper-middle-income
Uganda	Low-income
Ukraine	Lower-middle-income
Uzbekistan	Lower-middle-income
Vanuatu	Lower-middle-income
Venezuela, RB	Upper-middle-income
Vietnam	Lower-middle-income
West Bank and Gaza	Lower-middle-income
Yemen, Rep.	Low-income
Zambia	Lower-middle-income
Zimbabwe	Low-income

Table 4.
